# Are guidelines guiding? A mixed methods study examining the integration of ASCO fertility discussion guidelines in practice among oncologists and adolescents and young adults at an NCI-designated Comprehensive Cancer Center

**DOI:** 10.1007/s11764-025-01850-0

**Published:** 2025-06-19

**Authors:** Julia Stal, Serena Y. Yi, Charleen I. Roche, David R. Freyer, Sue E. Kim, Joel E. Milam, Gino K. In, Kimberly A. Miller

**Affiliations:** 1 Department of Population and Public Health Sciences, Keck School of Medicine, University of Southern California, Los Angeles, CA USA; 2 Cancer and Blood Disease Institute, Children’s Hospital Los Angeles, Los Angeles, CA USA; 3 Departments of Pediatrics and Medicine, Keck School of Medicine, University of Southern California, Los Angeles, CA USA; 4 USC Norris Comprehensive Cancer Center, Los Angeles, CA USA; 5 Department of Epidemiology and Biostatistics, Department of Medicine, Chao Family Comprehensive Cancer Center, University of California, Irvine, CA USA; 6 Division of Oncology, Keck School of Medicine, University of Southern California, Los Angeles, CA USA; 7 Department of Dermatology, Keck School of Medicine, University of Southern California, Los Angeles, CA USA

**Keywords:** Oncofertility, Cancer care delivery, Adolescent and young adult, Reproductive health, Guidelines, Mixed methods

## Abstract

**Purpose:**

This study aims to explore the integration of American Society of Clinical Oncology (ASCO) guidelines for fertility discussion in clinical practice.

**Methods:**

A concurrent triangulation mixed methods design was used. We recruited oncologists from an NCI-designated Comprehensive Cancer Center who treat adolescents and young adults (AYAs) at risk for infertility to participate in a semi-structured qualitative interview and conducted a thematic analysis. Simultaneously, self-report fertility-related data were collected from AYAs (age 18-39) diagnosed at the same institution via self-report survey and were analyzed using descriptive statistics.

**Results:**

Themes reported by oncologists (*N* = 12; 66.7% female, on average in practice for 14.3 years) included a lack of oncofertility-related training and limited knowledge surrounding fertility discussion guidelines. Those who were aware of guidelines stated that they informed their discussions. Oncologists’ perceptions of fertility discussion guidelines were largely positive, though reservations were expressed. Discussions were primarily informed by patient needs and research/literature, but seldom by oncologists’ explicit training or experience in oncofertility. Among AYAs (*N* = 58; 53.5% female, 35.1% Hispanic, on average 32.0 years at diagnosis), 82.3% had a fertility discussion, 62.6% of which occurred with their oncologist. Fertility discussions occurred at some visits (66.7%), and AYAs were very (39.4%) or moderately (27.3%) satisfied with counseling received. Components of ASCO guidelines most often discussed were the timing of preservation and consideration of individual factors in fertility preservation (66.7% each). Patient advocacy resources (33.3%) and informing them that their cancer history does not increase risk of cancer or birth defects in a child (30.3%) were least often discussed.

**Conclusion:**

Awareness of fertility discussion guidelines among oncologists was low, and more than half of AYAs reported only two components of ASCO guidelines were included in fertility discussions with their oncologists. Despite this, AYAs’ overall satisfaction with discussions was moderate to high, suggesting adherence to all guideline components may not be necessary for AYAs to derive benefit. While oncologists reported largely positive perceptions of fertility discussion guidelines, several shared that the guidelines themselves may hinder implementation if they do not capture diverse patient scenarios and/or are outdated.

**Implications for Cancer Survivors:**

Findings suggest a need to improve oncologists’ knowledge surrounding guidelines, refine recommendations to optimize oncofertility counseling, and subsequently improve their integration in practice to ensure AYAs are provided with desired and actionable information to support goal-concordant reproductive decisions.

**Supplementary Information:**

The online version contains supplementary material available at https://doi.org/10.1007/s11764-025-01850-0.

## Background

In the United States, over 84,000 adolescents and young adults (AYAs; 15–39 years of age) were estimated to be diagnosed with cancer in 2024 [1, 2]. Fortunately, advancements in cancer treatment have led to a 5-year relative survival rate of 86%, with cancer death rates decreasing by nearly 1% annually from 2013 to 2022 for AYAs [1, 2]. However, many effective treatment modalities, including chemotherapy, irradiation, and surgery, may be gonadotoxic and confer an elevated risk of subsequent infertility or subfertility [3–9]. While fertility preservation is highly complex with many factors conditioning its feasibility, only a small percentage of AYAs at risk for infertility are able to preserve fertility before cancer treatment [10–16]. As such, the reproductive age of AYA cancer patients makes comprehensive reproductive health care a unique and critical issue for this population.

Oncofertility clinical practice guidelines from the American Society of Clinical Oncology (ASCO), among other organizations, recommend that oncologists should discuss possible treatment-related infertility with all patients of reproductive age (defined by the American Society for Reproductive Medicine (ASRM) as females under mid-40s and males under 60 years) [6–9, 17, 18]. ASCO guidelines indicate that fertility discussions should include infertility risk, preservation options, referral to a reproductive specialist, costs, and patient-specific pregnancy risks [6–9]. Guidelines further indicate that such counseling should occur at the time of diagnosis to ensure the full range of preservation options remain viable for patients [8, 9]. These timely discussions are particularly relevant for AYAs with cancer as they are in the prime of their reproductive years and report fertility-related distress following their cancer treatment [19].

In many care settings, oncologists are the primary counselors and are expected to discuss fertility with patients [20–22]. However, the provision of oncofertility care varies across healthcare settings, such that oncofertility resources are often concentrated in academic medical centers where patients are more likely to be counseled on fertility [21, 23–25]. For example, most fertility preservation referrals originate from academic medical settings where most oncofertility patient navigators are employed [26, 27]. However, even in these highly resourced settings, unmet needs persist (e.g., one third of patients are not satisfied with the quality or length of oncofertility counseling received, among others) [28, 29]. Consequently, findings from one institution type may not be applicable to another. Therefore, a granular understanding of oncofertility care delivery within respective care settings is needed to inform nuanced interventions that can be sustainably implemented.

As reproductive health care is a critical component of oncologic care for AYAs, physician adherence to guidelines is important for comprehensive oncofertility care delivery. Without adequate counseling, AYAs are likely to lack knowledge about their reproductive potential, preventing them from mitigating adverse outcomes to fertility prior to treatment initation [30]. Notably, AYAs who report a fertility discussion also endorse greater reproductive concerns, suggesting a potential combination of heightened patient awareness of uncertainties surrounding future childbearing potential and the possible omission of valuable components in oncofertility counseling that guide patients’ decisions regarding their reproductive futures [31]. However, the content of fertility discussions has only recently begun to be explored in detail, and the extent to which ongoing discussions adhere to guidelines remains unknown [32].

The purpose of this study is to explore the integration of ASCO guidelines for fertility discussion in clinical practice. We sought to (1) qualitatively describe oncologists’ knowledge, perceptions, and uptake of fertility discussion guidelines when counseling newly diagnosed AYA cancer patients at an NCI-designated Comprehensive Cancer Center (academic medical center); and (2) quantitatively examine adherence to ASCO fertility discussion guidelines via self-report among AYAs newly diagnosed with cancer at the same institution. To our knowledge, these dual clinician-patient perspectives have not been reported, and such novel insights will inform needed interventions to ensure this at-risk population receives appropriate supportive care to support goal-concordant reproductive outcomes.

## Methods

**Fig. 1:**
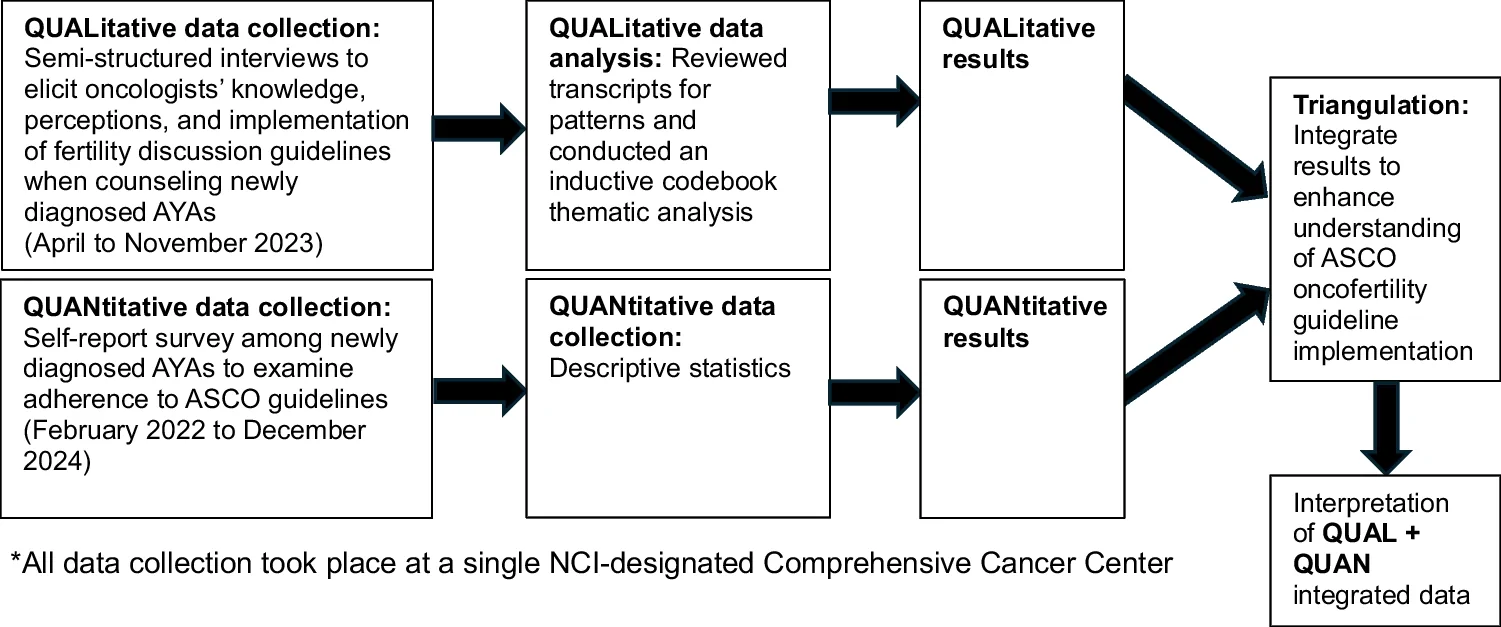
Concurrent triangulation mixed methods study design

This study uses a concurrent triangulation study design in which data are collected and analyzed simultaneously but separately (Fig. 1) [33–36]. We used qualitative research methods to describe oncologists’ knowledge, perceptions, and implementation of fertility discussion guidelines when counseling newly diagnosed AYA cancer patients and used quantitative research methods to examine adherence to ASCO guidelines in practice from the perspectives of AYAs newly diagnosed with cancer. Qualitative recruitment occurred from April to November 2023, and quantitative recruitment occurred from February 2022 to December 2024. Both participating oncologists and AYAs were recruited from a single NCI-designated Comprehensive Cancer Center (academic medical center). Of note, this care setting does not have a central oncofertility navigator, and cancer care teams address fertility on their own. To support patients and providers, this institution has an AYA program, which is a supportive care program that aims to conduct needs assessments and provide psychosocial and navigational support to AYA patients treated for cancer at this institution. Cancer treatment is delivered by the disease-specific team; therefore, this is a consultative supportive care service. Patients can be self- or provider-referred (i.e., by their cancer treatment team, nurse, and/or social worker) to the AYA program. For patients referred to the AYA program, a nurse navigator engages in a preliminary interaction regarding fertility with patients. The nurse navigator aims to assess patient awareness, knowledge, and potential interest in fertility preservation. If interested, female patients are referred to the gynecologic reproductive endocrinology team, and male patients are referred to the urology team. A social worker supports patients with financial navigation related to these referrals. Patients who are not referred to the AYA program do not consistently receive this fertility-related support.

### Qualitative data collection among oncologists

Study methodology and findings are reported in adherence with the consolidated criteria for reporting qualitative research (COREQ) 32-item checklist [37]. This qualitative research was conducted according to the guidelines of the Declaration of Helsinki and was deemed exempt and approved by the University of Southern California Institutional Review Board (IRB; UP-22–00847).

### COREQ Domain 1: Research team and reflexivity

#### Personal characteristics

The primary qualitative data collection team consisted of the PI (JS; an advanced doctoral candidate with research focus in oncofertility) and two research assistants (SYY and CIR, medical student and doctoral student, respectively); all are female, held bachelor’s degrees at the time of data collection, and had experience conducting cancer survivorship research. Data collection was overseen by the senior author (KAM) who is an experienced cancer survivorship researcher, and findings were refined with input from clinical co-investigators (DRF, GKI) who are oncologists treating AYAs and clinician-scientists.

#### Relationship with participants

The study team did not have a relationship with any participants, personal interests in the research topic, or assumptions.

### COREQ Domain 2: Study design

#### Participant selection

Recruitment was open until data saturation was reached, from April to November 2023. Oncologists employed at an NCI-designated Comprehensive Cancer Center in California who treat AYAs at risk for treatment-related infertility were recruited for study participation. This study only included oncologists as they are the treating physicians, are one of the preferred sources among AYAs for learning about late effects of treatment, and are widely believed to initiate fertility discussions [20, 21, 30]. Convenience and opportunistic sampling were used to identify eligible oncologists [38–40]. The PI (JS) contacted oncologists via email to recruit them for study participation. A follow-up email was sent to oncologists who were contacted for participation but did not initially respond. Prior to participating, oncologists received an information sheet with the elements of informed consent and verbally agreed to study participation and interview recording. After completing the interview, oncologists were provided with a $25 gift card and a list of oncofertility resources. In total, 27 oncologists were contacted, and 12 participated, resulting in a recruitment rate of 44.4%.

#### Setting and data collection

Oncologists were asked to complete a brief online survey using a Health Insurance Portability and Accountability Act (HIPAA)-compliant REDCap prior to the interview with demographic and fertility-related information. Collected demographic information included oncologist’s sex, race/ethnicity, specialty, and years of experience. Fertility-related information included whether they discuss fertility with their AYA patients (y*es*, *no*, *not sure*) and their level of confidence in doing so. Responses used a 4-point Likert scale and ranged from 1 “slightly” to 4 “completely” confident. Interviews were led by the PI (JS) with one co-interviewer present (SYY or CIR) via HIPAA-compliant Zoom.

A semi-structured interview guide designed to elicit oncologists’ subjective experiences discussing fertility with their AYA patients was developed with input from clinical co-investigators on the study (DRF) and is provided elsewhere [32]. Sample interview questions included, “What informs your discussions of fertility with AYAs?” and “How do you integrate existing guidelines in discussions of fertility with AYA patients?” After each interview, de-identified audio files were sent for verbatim transcription to an external service and were immediately deleted after transcripts were received. All study materials were stored on a secure server in accordance with HIPAA guidelines. The demographic survey took approximately 5 minutes, and the qualitative interview took under 60 minutes to complete. All study components were conducted in English.

#### Theoretical framework

In accordance with the Braun and Clarke (2006) six-phase method for establishing trustworthiness during thematic analysis, we reviewed transcripts for patterns and conducted an inductive codebook thematic analysis [41]. Data/thematic saturation was achieved through 12 interviews. This sample size has been evaluated and deemed sufficient for achieving data saturation among a homogeneous sample, such as in this study [39, 40].

### Quantitative data collection among AYAs

#### Study design, eligibility, and recruitment

Quantitative fertility findings were derived from a larger, longitudinal observational study examining social health and quality of life among AYAs [42]. Data were collected electronically via self-report REDCap survey. Eligible AYAs were diagnosed within the past 3 months at age 18 to 39 years and with de novo cancers prototypical for AYAs, stage I–IV, and an expected survival of over one year at the time of diagnosis. The parent study contained four waves of data collection, with the first wave collected at recruitment when AYAs were within 3 months of diagnosis. Fertility-related information was collected during the second wave; thus, AYAs were within 6 months of diagnosis when responding to these items. For the parent study, AYAs must be diagnosed at one of three California medical centers; however, for the purposes of this cross-sectional study, only AYAs who were treated at the same NCI-designated Comprehensive Cancer Center as the oncologists who participated in the qualitative portion were retained in the analyses. This study was conducted according to the guidelines of the Declaration of Helsinki and was approved by the University of Southern California IRB (UP-20–00993). A partial HIPAA waiver was granted to identify patients in the electronic medical record (EMR). The study was approved for written informed consent from participants for study participation.

AYAs identified via EMR and who were eligible for participation were contacted by a recruitment specialist. Signed informed consent was obtained online via REDCap. For the parent study, participants were compensated $50 for completing each assessment period (four study waves in total) plus a $50 bonus for finishing all four waves of assessments for a total possible compensation of $250. Thus, for completing the wave of the survey that contained the fertility items described in this manuscript (wave 2), participants received $50 (in addition to receiving $50 for previously completing wave 1 of the survey). Additional details regarding study design, eligibility, and recruitment for the parent study are described elsewhere [42].

### Measures

#### Fertility-related information

AYAs were asked to report their reproductive history before their cancer diagnosis (*previous live birth*, *previous miscarriage*, *normal menstruation*, *history of infertility*, *adoption*), if they experienced infertility as a result of their cancer treatment (*yes*, *no*, *not sure*), and if their monthly menstrual cycle (period) stopped after cancer treatment (*yes*, *no*, *not sure*, *not applicable* [*I didn’t have a menstrual cycle* (*period*) *before cancer*]). Those who responded that their monthly menstrual cycle stopped were asked to indicate if it returned after treatment (*yes*, *no*).

#### Fertility discussion

AYAs were asked if they were informed of the impact of cancer treatment on their fertility prior to beginning treatment (*yes*, *no*, *not sure*, *not applicable [i.e., treatment would not impact fertility, post-menopausal, not interested in having children, etc.*)*.* AYAs who reported that they had a fertility discussion were asked to report the frequency of discussion (*at every visit*, *at a majority of visits*, *at some visits*), their level of satisfaction with the discussion received (*very satisfied*, *moderately satisfied*, *neither satisfied nor dissatisfied*, *moderately dissatisfied*, *very dissatisfied*, *not applicable* [*I did not need fertility preservation counseling*]), who informed them of the impact of cancer treatment on their fertility (check all that apply: *medical oncologist*, *physician assistant or nurse practitioner*, *primary care provider*, *gynecologist*, *social worker*, *other* [*free response*]), and the frequency (*always*, *usually*, *sometimes*, *rarely*, *never*) with which each of the following items (disaggregated components of guideline-concordant fertility discussions as recommended by ASCO) [6–8] were included in their discussion(s): (1) individual factors such as disease, age, treatment type and dosages, and pre-treatment fertility; (2) considering your options as soon as possible to increase the likelihood of success; (3) if you are female, timing of fertility treatments based on your menstrual cycle (period); (4) there is no increased risk of cancer returning because of preserving fertility and pregnancy; (5) a history of cancer, cancer treatment, or fertility treatment does not increase the risk of cancer or birth defects in your child, except in special circumstances; (6) offered a referral to supportive counseling prior to treatment with adequate time to use these resources; and (7) provided with information to patient advocacy resources (for example, FertileHope, Livestrong, the Susan G. Komen® Breast Cancer Foundation, and the Colon Club).

## Data analysis

### Qualitative data analysis

#### COREQ Domain 3: Analysis and findings

##### Data analysis

Frequencies were used to describe demographic characteristics of oncologists who participated in the study, to quantify the frequency of fertility discussion with AYA patients, and to determine their level of confidence discussing fertility. Regarding thematic analysis, we (JS, SYY, CIR) first took theoretical/observational notes (i.e., memoed) on initial interviews from which we generated an initial set of codes. Then, we applied the initial codes to new interview transcripts. The coding scheme was developed through an iterative process. We used frequently voiced themes to develop codes, including themes mentioned in interviews that were not specified in the original interview guide. We reviewed codes and themes as they were developed, considered alternatives, and revised them before arriving at a set of thematic codes. The codebook was subsequently refined and adapted as needed. Transcripts and codes were then uploaded and analyzed using ATLAS.ti (Version 22.0.1), a computer-assisted qualitative analysis software program, for line-by-line manual coding by the data collection team (JS, SYY, CIR). Each document was coded by two researchers, and disagreements were resolved through group discussion until consensus was reached.

##### Reporting

Major themes are italicized in the results section of this manuscript. Supporting quotations from various study participants are described in the text. Additional quotations comprising developed themes are provided in tables as described below.

#### Quantitative data analysis

Descriptive statistics (i.e., frequencies) were used to characterize the sample and responses to fertility-related items. Analysis was performed using Stata software version 15 (StataCorp, 2017).

### Data integration

Following concurrent data collection and analysis, each set of data received equal focus and was integrated following a triangulation approach to understand the integration of ASCO fertility discussion guidelines in practice (Fig. 1). To protect oncologists’ anonymity and refrain from scrutiny of individual care delivery, we do not pair qualitative responses among oncologists to patient quantitative responses. Rather, we use findings to expand upon one another and to better understand how ASCO guidelines are or are not used in practice. Results are presented using a contiguous approach, in which qualitative and quantitative findings are reported in different sections to facilitate interpretation, relay nuances in our findings, and illustrate how each data collection method contributed to the overall comprehensive understanding of our study purpose.

## Results

### Qualitative findings

Participating oncologists (Table 1; *N* = 12) were 66.7% female, 41.7% of White or Asian race (each), and reported a mean time in practice of 14.3 years (SD=6.7; range: 8–29). Oncologists included one breast medical oncologist, two genitourinary medical oncologists, two gynecologic oncologists, three sarcoma and melanoma medical oncologists, one urologic oncologist, one solid tumor/cutaneous oncology medical oncologist, one neurooncologist, and one gastrointestinal medical oncologist (participating oncologists are referred to as “medical oncologist” for all specialties, with the exception of gynecologic oncologists who are referred to as such in an effort to preserve anonymity but relevantly distinguish between specialties and findings). Prior to the qualitative interview, all oncologists endorsed discussing fertility with their AYA patients via self-report survey, and were, on average, “somewhat” to “fairly” confident discussing fertility (mean=2.9, SD = 0.9; range: 1–4, where 4 is most confident). In total, 50% of the participating oncologists reported that they were “fairly” confident discussing fertility (n=1 reported being “slightly confident,” 2 reported being “somewhat confident,” 6 reported being “fairly confident,” and 3 reported being “completely confident”). The patient population seen by participating oncologists is reported elsewhere; oncologists reported that AYAs comprise 15 to 40% of their practice [32].

**Table 1:** Qualitative sample characteristics among oncologists

	***N* (%) or *M* (SD)**
**Sex**	
Female	8 (66.7)
Male	4 (33.3)
**Race/ethnicity**	
White	5 (41.7)
Asian	5 (41.7)
Hispanic	1 (8.3)
Other	1 (8.3)
**Mean years in practice**	14.3 (6.7)
**Discuss fertility**	12 (100.0)
**Confidence discussing fertility**	2.92 (0.90)
Slightly (1)	1 (8.3)
Somewhat (2)	2 (16.7)
Fairly (3)	6 (50.0)
Completely (4)	3 (25.0)

*All* *medical* *oncologists, except gynecologic oncologists, reported that they had no formal training concerning fertility discussions, nor did other members of their multidisciplinary care teams (Supplemental Table* 1).﻿ Gynecologic oncologists were the only providers that indicated some training discussing oncofertility. One gynecologic oncologist reported, “Just the physicians [have training], like me and my partners if they were covering for me, but not any of the other nurses or mid-level providers.” By contrast, one medical oncologist mentioned,

“I don’t know that anybody truly has training. It’s just me and her [nurse practitioner]. I don’t know that she’s done any extra training for it in her studies. As far as my studies go, there’s not something that’s taught to us in med school as far as chemotherapy infertility other than to say that it causes infertility. So, I don’t know there’s any special courses that at least I haven’t gone through.” (Medical oncologist)

Several expressed that their training was informal and consisted of gaining experience over time or by collaboration and crowdsourcing with mentors or colleagues. One medical oncologist mentioned,

“Just by experience and having conversations with other practitioners. I always get tips, or someone says ‘Oh, I found this great resource.’ So just discussions with other people, collaborations with other specialties.” (Medical oncologist)

Another medical oncologist added,

“Training? Gosh. I don’t think anybody’s had any training. Specific training for reproductive health or fertility, we just learn as we go.” (Medical oncologist)

Lectures or rotations on oncofertility during training were rarely reported. Some mentioned that their medical education emphasized the pharmacologic effects of cancer therapies but did not include fertility discussions. One stated,

“(Laughs) That’s a good question... I certainly don’t have any certifications or licenses specific to it. I do have training regarding side effects and potential side effects related to treatments. So, in that regard, I would say that I have some, but I think if it’s anything more specialized than that, no we don’t have anybody, not directly on my team at least.” (Medical oncologist)

Lack of oncofertility training among oncologists did not appear to be offset within their team, as only one oncologist reported that only social workers had formal training in discussing fertility with patients:

“I don’t think any of us have formal training. I guess in my fellowship training we certainly had lectures and such, but I don’t think…. Do you mean our nurses and our PAs? I don’t think they get any formal training. Though, of course, the social workers do and we used to work closely with our AYA social worker who left [institution] a few months ago. I think they just hired a replacement, so I’m sure we’ll be working closely with [them]. I’m sure they have formal training there.” (Medical oncologist)

*Many oncologists reported that they were* *not aware of* *specific guidelines for* *fertility discussion and/or* *had not used them (Supplemental* *Table* 2). When asked about guidelines for discussing fertility, one oncologist reported:

“I don’t know. [Laughs] Maybe you could tell me. I’m not aware of any guidelines on fertility discussions. Maybe it’s more prevalent in the GYN oncology world but for medical oncology I’m not aware of guidelines for fertility discussion.” (Medical oncologist)

Notably, one provider mentioned that NCCN guidelines inform their discussions of fertility, but later reported that they were not familiar with these guidelines beyond needing to mention fertility:

“Not anything above what I’ve mentioned. Things that are major announcements at meetings, or articles that come out that get a lot of press. NCCN guidelines are always useful…I’m not sure of the guidelines that are specifically in place, other than that it’s a good thing to mention and to offer counseling on it. I’m sure there are guidelines, but I’m not familiar with them.” (Medical oncologist)

When asked about what external clinical practice guidelines exist for fertility discussions, oncologists identified the following guidelines: NCCN, ASCO, American Urological Association (AUA) Testicular Cancer Guidelines, American Society of Hematology (ASH), European Society of Medical Oncology (ESMO), Society for Immunotherapy of Cancer (SITC), Society of Gynecologic Oncology (SGO), and the American Congress of Obstetricians and Gynecologists (ACOG; see Supplemental Table 2). A few oncologists who reported that they were not aware of guidelines for discussing fertility were able to speculate that large, well-known oncology clinical practice organizations must have developed them. For example, one stated, “I think that there are some NCCN guidelines related to this, but I’m not 100% sure” (Medical oncologist). Another added,

“I’m sure there is stuff that ASCO has. I’m sure there are things that NCCN has…Honestly, I have never read them. So, I am going to be totally honest. I assume they exist, but I have never seen them.” (Gynecologic oncologist)

Some oncologists explicitly reported that their limited knowledge of oncofertility hindered their fertility discussions. For example, one oncologist stated, “I don’t know specifics in terms of cost, so I can’t be the one to do it,” implying that they are unable to counsel patients on the cost of fertility preservation (Medical oncologist). Another echoed this, stating,

“I don’t know the data all that well either, so I give them a rough estimate, but I think that when I’ve searched in the past, the studies that I’ve seen aren’t really specific to any chemotherapy drugs, so sometimes patients will say, ‘What percentage do I have of getting pregnant after if I do chemotherapy?’ And I haven’t really seen excellent, clear, randomized trials obviously, or really great retrospective studies. So, I give them my estimate with the limitation that I think there’s not great data to support it, but to understand with the assumption that it’s less than before.” (Medical oncologist)

*Oncologists’ perceptions of fertility discussion guidelines were* *largely positive, though some reservations* *were expressed. However, limited knowledge of guidelines hindered oncologists’ ability to describe how actionab*l*e they perceived components of guidelin*es *to be (Table* 2). All oncologists positively described guidelines as helpful, either because they are a good resource, useful for care recommendations, as a reference, template, or reminder, or for establishing a minimum standard of care and consistency across providers. Several described oncofertility guidelines as “necessary” and some as “adequate” for purposes of guiding discussions. One oncologist added that guidelines are helpful but it is important to have personal experience discussing fertility. They elaborated,

“I think they’re helpful from the informational standpoint. I think though, medicine is an art and so, guidelines are guidelines. They’re helpful at dispensing information, but I think you really have to have practice in delivering this. In medicine we say watch one, do one, teach one, and so, I think absorbing somebody deliver the news, empathizing with the patient, I think those are very, very helpful for observers who are just starting their practice on infertility counseling. Eventually they develop their own style and art, if you will.” (Medical oncologist)

**Table 2:** Oncologists’ perceptions of guidelines for fertility discussion

	**Quote**	**Oncologist**
**Positive perceptions surrounding guidelines**		
**Necessary**	“I think they’re always necessary, if not anything more than just to remind practitioners that this is important. A lot of the time we fixate on treatment and response and may not consider this aspect of someone’s life. So, whether or not you strictly follow guidelines, I think it’s important at least for review and to remind us all this is something we should focus on.”	Medical oncologist
	“I definitely think they might be necessary.”	Gynecologic oncologist
	“I think they’re necessary because this is a very sensitive topic and delivering it in a way that is informative should be the focus of the discussion and that’s what these guidelines do, is they give you good information. They give you guidelines and options for patients as well. So that really helps balance that hope versus trauma in discussing infertility with patients. So those guidelines really give you a nice way of doing things and really it should be patient focused as much as possible.”	Medical oncologist
	“I think they are (necessary). I think it would be helpful to have clear guidelines.”	Medical oncologist
	“I think guidelines are necessary because we need to mention these things because some providers often forget. They think it can be dealt with afterwards and by then, it might be too late. If the patients start treatment and they have subfertility and afterwards cannot get pregnant, something could have been done prior. If you bank sperm, prior to initiation of treatment, then you have the optimal best chance of getting pregnant. So, I think these guidelines are necessary so that practitioners are aware that this should be something they discuss beforehand.”	Medical oncologist
	“I think guidelines are necessary…But I know that taking into account the medical system, and how other guidelines are set, sometimes the guidelines become a minimum, that, at the very least, we should be doing these things. I think, really, guidelines should strive to be something that we work on achieving, something higher, something better that we can achieve for all of our patients, just to make sure that they have equitable care, no matter where they are, or who they’re being treated by.”	Medical oncologist
**Standard of care**	“I mean it’s pretty standardized.”	Medical oncologist
	“I think they establish what many people will view as being the standard of care and what’s a minimum level of accepted requirement that we should make sure that the patients are aware of.”	Medical oncologist
	“It does provide them a framework to at least make sure it’s included and hopefully at least sets a bare minimum of that task people should be adhering to.”	
	“They help to set a standard for providers everywhere. I would hope that that standard is of a high level.”	Medical oncologist
	“Guidelines are always nice to have to create a standard of care and on a topic that’s important like this, I think it’s important that everybody addresses it. Hopefully all doctors are aware of the importance of fertility in young patients. I think most people are, but guidelines make it a level playing field for all patients. So, I think it’s important to have guidelines, but on a topic as important as this, I would hope that every doc is already addressing it and we don’t need guidelines to tell docs what to do.”	Medical oncologist
	“There’s a consistency, right? I think across providers, I’ve definitely had patients come for second opinions about this, when they’re trying to figure out what to do and the guidelines are a static thing, for the most part, within that short period of time that they apply to that patient. So, to be able to fall back on them and quote them is helpful and I think especially if patients are wanting to do something that is not within the guidelines that they understand that it’s not just me and what I know and what I’m recommending, there’s a lot of data and experience behind this and to me, it just carries more weight.”	Gynecologic oncologist
**Reference**	“I think guidelines are really great for being a point of reference, if you have questions about what to do about certain scenario. So, I appreciate guidelines as a resource, but not as a requirement.”	Medical oncologist
	“I think they’re a great resource, similarly, it’s a great place to go. You know who’s curating them, it’s a very trusted source. Great place, they’re very nicely outlined and updated so I enjoy using them.”	
	“Just as a template, same way that guidelines are there for all of our other treatment decisions. These are the recommendations, this is where the data and the evidence lies that helps us to decide what to do next.”	
	“It’s a reference basically…It really comes down to remembering to discuss timely initiation of the discussion and an understanding of the options.”	Medical oncologist
	“I think that how I view these guidelines and how other people may view them is very different. I think that for many people, these guidelines are sort of a cheat sheet in terms of what you do in this situation. The way I view these guidelines is they provide a template, a guideline that provides some sort of minimal standard of care, which everybody should follow, but in my feeling, if the patient has more specific factors that warrant further individualized approaches, I will tend to build on those as necessary. Just because they meet some minimal level of care does not always necessarily mean that they’re adequate, in my opinion.”	Medical oncologist
**Helpful for providers who are unfamiliar with AYAs or reproductive health**	“I think if you are uncomfortable with them (fertility discussions) or if you’re unsure what to do, if there’s chemotherapies that may be overly protective, or if you have to think about things differently than you normally would, then I think that they would be helpful for that reason.”	Gynecologic oncologist
	“It gives you a map to start off with and I think there’s a lot of folks that have never seen an AYA patient, so they never talk about these issues. So, I think for those folks, 20% of my practice is AYA, so I’m very familiar with it, but I can see how those that just see prostate cancer patients, like 70 years old and older, maybe not know how to interact and in fact, they may not know how to interact with young patients, period. Right? So, the guidelines would be helpful, but I think maybe more of a global this is how you deal with young patients’ curriculum would be more helpful for them because it’s not so much an issue of infertility. It’s an issue of dealing with a young patient who has their whole life ahead of them and has all these unique identity and psychosocial issues. So, yeah, as part of a larger curriculum in dealing with young patients, AYA patients, I think it would be useful.”	Medical oncologist
	“It’s nice for if there are some people who don’t regularly have these conversations and don’t regularly think about having these discussions.”	Medical oncologist
**Helpful**	“I think they’re a helpful tool but as with any guidelines, it’s not the final authority. I think like with all cancer care, it’s a multidisciplinary approach. So having a team that has good communication is more important than relying solely on guidelines, but I think they’re there, it’s a good reference to have.”	Medical oncologist
	“I think that they are helpful. Obviously like everything else in our field it’s not something that you can use 100% because patients are different, they don’t always fit the textbook, they have different preferences, different everything, but I think it’s helpful as a reminder, some guidance to use with caution, but I think it’s a good idea to have these guidelines.”	Medical oncologist
	“I think it’s also helpful because it helps obtain authorization. So oftentimes insurance companies will use these guidelines to justify cost reimbursement or provide authorization. So, I think from that standpoint, calling it a standard of care also justifies the cost. I think that’s an important thing.”	
**Adequate**	“Well, they’re guidelines. I think they’re adequate and I think there’s been a lot of AYA-centric guidelines now for infertility and I think that’s encompassed within those NCCN guidelines as well as the ASCO guidelines. I think they’re helpful from the informational standpoint. I think though, medicine is an art and so, guidelines are guidelines. They’re helpful at dispensing information, but I think you really have to have practice in delivering this. In medicine we say watch one, do one, teach one, and so, I think absorbing somebody deliver the news, empathizing with the patient, I think those are very, very helpful for observers who are just starting their practice on infertility counseling. Eventually they develop their own style and art, if you will.”	Medical oncologist
	“I think they’re adequate. I think they can be helpful. I think it’s a nice summary to refer to the different options, but every patient is very unique, so it has to be individualized to that patient. I mean I think they’re all just outlining the different options, and I think that that’s helpful.”	Medical oncologist
**Negative perceptions of guidelines**		
**Too stringent or may not capture varying patient situations**	“I think they would only hinder if we were too strict about working within those. If there’s a patient who’s 41 and not 40, and still wants to consider them, we can be flexible as a physician to make the call. You know, this is a patient who has been trying to have children for 10 years and, X, Y and Z. You have to take into consideration the whole patient. So, you can’t be so stringent with regard to the way the guidelines are written.”	Medical oncologist
	“I think they (guidelines) could be better structured with better scientific literature to support what they do and what they accomplish. I think it’s still a bit nebulous when it comes to what they do and what they achieve and because of it, it’s difficult for the provider and also the patient to understand when things should be brought up. So, they probably need to be fleshed out and developed a little better. Does the patient want to have a family this year? Does the patient want to have a family in 10 years? There are all kinds of little nuances. Does the patient want to have one child? Does some patient want to have multiple children? These are things which the guidelines I don’t think touch on at all. What’s the patient’s current age? There’s a lot that I think probably is still missed on. It’s rather simplified in the sense that it’s like okay, the patient may lose fertility, they may not, make sure they understand that. With these diseases, this may be the percentage of it happening, you can still have children afterwards. Probably needs to be gender specific, and then maybe even tumor specific as well, and then obviously when it comes to medical therapies that we as oncologists prescribe, it needs to be probably treatment specific too. So yeah, I think the guidelines just need to be more robust and then we would probably have greater utility for them and rely on them more.”	Medical oncologist
	“It’s important to remember that patients have free will and they need to be part of the discussion of their care, and they need to be free to make decisions on their own. So even if you think that’s not the best decision or that’s not what the guideline says you should do, they have the right to make those decisions and there’s some people that may make decisions that you think are not the smartest decision. Like we had a patient who had ovarian cancer, and she refused to remove one of her ovaries and she had bad epithelial ovarian cancer, and we were like you need to take both. This is not one where you can spare the ovary, and she refused. I’m not going to take out an ovary that she says she won’t take out.”	Gynecologic oncologist
	“I think there’s always case-by-case basis and specific patient preferences. We might have a patient whose cancer is a little too high risk for the fertility-sparing option, but they feel very adamantly that that’s what they want, and they won’t proceed with the more aggressive treatment if it means they’re going to lose their fertility and sometimes there may be some room there to not adhere to the guidelines or to go by lower-level evidence that isn’t necessarily in the guidelines but has maybe been small studies that have tried certain things in that instance or something like that.”	Gynecologic oncologist
**Outdated or do not capture ongoing developments**	“Guidelines sometimes stick you to a very straight pathway and it may be that there are new and developing things that are coming out that wouldn’t necessarily be included in the guidelines.”	Gynecologic oncologist
	“Some of these guidelines are quite up-to-date and very informative and some of them are quite outdated and not so helpful. So, I do try to look at more than one set and try to pull out what I think are the best aspects in all of them.”	Medical oncologist
	“I think that a lot of the guidelines that some of these societies provide were maybe developed decades ago and as oncology is a field that’s becoming increasingly more specific and personalized, individualized, I think some of these guidelines are actually becoming quite outdated at an even faster pace. So yeah, I think that some of them definitely need to be more modernized. So, for example, if I’m only adding guidelines based on patients developing infertility related to chemotherapy but I’m only using immunotherapy, then those guidelines, how much are they helping me?”	
	“Well, they certainly can hinder if they’re out of date and they’re recommending unnecessary things, which maybe are not relevant to a certain patient, so depending on the therapy, for example. Or if they potentially put patients under a lot of additional stress, that’s not really helpful to them in their cancer care and then that can potentially be a detriment as well.”	
	“I think patients can sometimes be a little afraid to, and providers, to not adhere to the guidelines if guidelines exist, even if there’s a lot of experience or data that suggests that you could do something else. The guidelines seem to lag behind a little bit, in terms of what data is out there. If there’s this guideline and they know about it, and I’m saying like, ‘Oh, but we could do something else,’ then I’d be more hesitant to do that even if it’s a totally reasonable option.”	Gynecologic oncologist

Another oncologist added that guidelines can be helpful for obtaining insurance authorization, stating that they can be used to “justify cost reimbursement or provide authorization” (Medical oncologist). Some oncologists emphasized that oncofertility guidelines are particularly helpful for providers who are unfamiliar with the AYA population or with reproductive health. One mentioned,

“I think if you are uncomfortable with them [fertility discussions] or if you’re unsure what to do, if there’s chemotherapies that may be overly protective, or if you have to think about things differently than you normally would, then I think that they would be helpful for that reason.” (Gynecologic oncologist)

Oncologists described ways in which oncofertility guidelines may hinder care, reporting that guidelines that are too rigid may impact care and may not capture varying and unique patient situations. Some also mentioned that guidelines that are outdated or that do not capture ongoing developments may hinder care. One stated,

“I think that a lot of the guidelines that some of these societies provide were maybe developed decades ago and as oncology is a field that’s becoming increasingly more specific and personalized, individualized, I think some of these guidelines are actually becoming quite outdated at an even faster pace. So yeah, I think that some of them definitely need to be more modernized. So, for example, if I’m only adding guidelines based on patients developing infertility related to chemotherapy but I’m only using immunotherapy, then those guidelines, how much are they helping me?” (Medical oncologist)

*Fertility discussions were primarily described as informed by patient-level factors and existing research/literature, but seldom by provider experience or training (Table* 3)*. All oncologists who were aware of guidelines reported that they referred to them for their discussions.* When asked what informs their fertility discussions, nearly all oncologists reported that patient-specific factors inform their counseling. These included the patient’s treatment plan, relationship status, family building goals, sex, age, readiness to discuss fertility, desired knowledge, medical history, socioeconomic status and background, and genetic syndrome status. One oncologist added,

“Even some of our really young [patients] are really not thinking of having kids right now, but trying to remind them that, ‘Yeah, maybe that’s not your mindset right now, but think 10 years in the future. If you’re interested in having that option, then I would recommend sperm banking and going from there.’” (Medical oncologist)

**Table 3:** What informs fertility discussions from the perspectives of oncologists

	**Quote**	**Oncologist**
**Patient-level factors**		
**Patient readiness**	“There are times when patients are saying that they’re not ready to commit to doing something like an egg harvest or intervening on their fertility options right now, but let’s say a couple of years into their treatment, because some of our treatments last for five to ten years, they’re interested in discussing it. So, at that point we say, ‘Okay, there’s a risk of pausing your treatment right now but it can be done,’ and we discuss the risks.”	Medical oncologist
**Family building goals**	“Just seeing where they are in terms of whether or not they want to have children at all, or if that’s something they’re considering. So, just asking those questions and where that leads us.”	Medical oncologist
**Age, current children, relationship status**	“Age and whether or not they have children already…whether or not they have a partner in the room with them would probably lead to a conversation about safe practices while on treatment.”	
**Treatment plan, age, family building goals**	“It really depends on what exact treatment we’re recommending. So, say we’re recommending something that at the most extreme, would remove their fertility entirely, like radiation or a hysterectomy or an ovariectomy. It’s entirely dependent on the type of cancer they have and what other treatments are available. So, let’s say somebody just has an ovarian cyst but it’s on both sides and it’s very large and the patient is young and we don’t know whether it’s cancer or not. We’ll automatically talk about what does it look like to remove those ovaries. Should we try to do a cystectomy? What is the risk of doing a cystectomy as opposed to removing, say, both ovaries? So, you have to talk to the patient to figure out what it is that they want and what their desires are.”	Gynecologic oncologist
**Treatment plan**	“I think that you need to verify that (they don’t want fertility) with every patient and we verify that even in patients who are undergoing procedures that might affect future pregnancy. Not even like fertility, but future pregnancies. So, like when we do LEEPS (loop electrosurgical excision procedure) and cones (conization, cone biopsy) and cervical biopsies to remove precancer from the cervix, that is always a discussion that I have with them. This is going to affect future pregnancy, and you should know that.”	
**Age, treatment plan**	“You can’t just assume that because somebody is 40 or 42 that they don’t want to have babies anymore, and honestly, in today’s world, women can have babies up until age 50. More than that if they use somebody else’s eggs or if they have their eggs in storage. So, I think it’s not safe to assume that taking out their uterus is just cool because they’re like 45 and that they won’t want to have fertility.”	
**Treatment plan**	“If I know a patient is going to be fertile with therapy like 80% or greater, yeah, I’ll make it a little bit lighter of a discussion about fertility, but those folks that have over 50% who are getting intense therapy and have a high chance of infertility, I will talk about that a little bit more deeply.”	Medical oncologist
**Desired knowledge**	“We ask them exactly how much they know. Actually, we do ask them how much they want to know as well. Most of them come out there and say I want to know everything. So, we usually start with that as a rubric. How much do you know? How much do you want to know? Then we go from there.”	
**Treatment plan**	“If they’re going to receive any cytotoxic therapy or any therapy from me that’s going to decrease their chances of fertility, then I would discuss it. Likewise, if they are planning for some urological procedure that would impair their ability to have children, such as an orchiectomy, then I would discuss that with them as well.”	Medical oncologist
**Current children, relationship status, family building goals**	“I start with asking, do you have any children currently? Then, based on their answer, do you have a partner? Are you interested in having children in the future? Then, based on their response, letting them know, regardless, how their treatment would impact their ability to bear children in the future and then offer fertility resources, fertility preservation. Even some of our really young (patients) are really not thinking of having kids right now, but trying to remind them that, ‘Yeah, maybe that’s not your mindset right now, but think 10 years in the future. If you’re interested in having that option, then I would recommend sperm banking and going from there.’”	
**Treatment plan**	“Usually if the plan is for chemotherapy, then we have to have a frank discussion that fertility can be impacted and the different options. Usually young women are referred to a fertility specialist to talk about different options and then we work with that fertility specialist to say, okay, here’s a prognosis for the patient, here’s the regimen that we’re planning. Then we discuss what options would be suitable for that individual. Of course, that’s primarily in the setting of patients who are undergoing curative intent treatment because their prognosis is favorable in the long term. Unfortunately, if patients have a more guarded prognosis and they have very symptomatic disease and they need to start therapy urgently, then for young women we may not have the time to preserve fertility as best as we can. Unfortunately, because of the guarded prognosis, it may not be an option.”	Medical oncologist
**Medical history, treatment plan**	“What informs my discussion is the history and physical of the patient, sometimes they’ve already had orchiectomy in the past and they will 100% be infertile. So, I have to discuss that with them and that there’s no other way to have children in the future obviously, but that’s a rare situation. Most of the time they have one testicle, but if they have a semen analysis available, it’s rare that they do when they come to me, but sometimes it’s possible, so that comes into the discussion. If they have a completely normal semen analysis, then we tell them you may not need to bank because everything looks normal with the one testicle, but it’s up to you. You’re undergoing an orchiectomy; you may have a decrease in the quality and quantity of the sperm afterwards with one testicle. So again, it’s always best to bank prior and I would say my patients, probably 20% or less will actually bank.”	Medical oncologist
**Socioeconomic status, current children, family building goals**	“Generally when I first meet the patients, it’s important for me to understand their socioeconomic status, their background, where they’re at in life in terms of their career goals, et cetera, and one of the goals obviously also is where they’re at in terms of trying to start a family and I think it just depends on how much further we need to go into that discussion at that point. If they already have a family and it’s not of too much interest to them, then we don’t dwell on that, but if there is a need for further discussion about it, then that’s where it starts.”	Medical oncologist
**Treatment plan, family building goals**	“I think the first question is what types of treatments are being considered for the patient. This is assuming that they are certainly concerned about fertility. So, the first question would be what treatments do we recommend, and then which of those treatments are potentially going to affect fertility. Some patients may require very minor treatment, maybe a quick procedure that will have zero impact, whereas some might get very aggressive therapies that have potential for serious side effects…So on the one hand, one patient may need very minimal care and another may need something pretty exhaustive and they want to get second opinions or get plugged in with other specialists if there are particular concerns about it.”	
**Family building goals**	“It just has to do with making sure I understand what the patient’s goals are, making sure I listen to them and give them adequate time to voice their concerns and their priorities. I always make sure the patient has plenty of time to ask their own questions. Long after I’ve explained everything that’s my usual topics of discussion, I always try to say to them, ‘What else do you need?’ So, I feel like if that’s really a priority for them, a lot of times that will usually be where it shows up in the conversation.”	
**Family building goals, relationship status**	“If they’re done childbearing, then it’s not that big a deal. But if they’re not even married yet, then I would say it’s a big deal. So, if they are interested in childbearing, we try to get them over to a fertility specialist immediately.”	Medical oncologist
**Family building goals**	“I would start by assessing someone’s desire to have more kids. If they aren’t sure or, well, I guess regardless, we’ll ask them if they have any idea of timeframe when they want to have their kids or events like that. Like, ‘Do you have a current partner that you want to have kids with?’ or specifics about that to get a sense of what they have thought about or what their desires are. Then, I will inform them about what their treatment options are that are fertility sparing, if there are any, and discuss if that’s something that they would want to pursue and if that has any impact on their cancer outcome, to do more fertility-sparing options.”	Gynecologic oncologist
**Treatment plan, age, sex, family building goals, genetic syndromes**	“Mainly anticipated infertility from treatments, their age, perhaps sex, if they have children or would like to have children in early discussions, and I think that’s mainly it. Of course, if they have genetic syndromes, then we make sure to have genetic counseling involved because that can also impact their fertility or planned fertility or making those decisions.”	Medical oncologist
**Existing research or literature**		
	“Things that are major announcements at meetings, or articles that come out that get a lot of press.”	Medical oncologist
	“The literature. Although I will say the literature is not always clear as to the percentage that will become infertile with certain types of therapy.”	Medical oncologist
	“Either individuals or seeing anything in the literature.”	Medical oncologist
	“In my practice, a lot of the drugs we use are actually fairly new and sometimes there’s actually unknowns regarding fertility. So, we sometimes have to make sure that the patient knows what the current literature says and then try to help them devise a good strategy when it comes to that.”	Medical oncologist
	“If I’m planning on having the discussion with each patient and then somebody demonstrates interest, I go back and again do a bit of a literature review. From a fertility standpoint for chemotherapy, you usually have to do an updated literature review.”	Medical oncologist
**Fertility guidelines**		
	“Occasionally, but not often (references guidelines in discussions).”	Medical oncologist
	“There are certain guidelines, depending on what you’re talking about. So, there’s guidelines for ovarian conservation in ovarian cancer. There are guidelines for ovarian preservation with endometrial cancer, and so, there’s different, you know, if I have a question about something, or if I was like, I don’t know, how old should I let this person keep their ovaries, right? There are instances where, say, you take out the uterus and leave the ovaries behind and the age of the patient just tells what you should do and what you shouldn’t do. So, there’s guidelines that kind of help us deal with that based on risk statistics.”	Gynecologic oncologist
	“We routinely use those (guidelines) to guide us. But generally, we’re guided down now. We have a new social worker coming in, so we’re going to probably refer him into the guidelines so that they’re informed.”	Medical oncologist
	“You may look at guidelines, for example NCCN guidelines, or ASCO guidelines kind of help.”	Medical oncologist
	“I think we definitely use guidelines that are provided by the American Society for Clinical Oncology and then I combine that with other resources that I have relevant to specific drugs that I use. So, for example, I use a lot of immunotherapies, and in that context, we follow what guidelines they provide. For some of our skin cancer patients or our AYA patients, we’ll sometimes try to look for resources there as well.”	Medical oncologist
	“I definitely incorporate guidelines from NCCN and SGO. A lot of them it’s just sort of best practice for GYN cancers. For instance, for endometrial cancer, there’s definitely some more kind of verified guidelines about doing hormonal therapy, which can be fertility sparing, versus surgery, and there’s more guidelines about at what age to take out the ovaries or when you can commit to leaving them. I think within each cancer site there’s often guidelines that weigh into that.”	Gynecologic oncologist
**Experience or training**		
	“Well, experience.”	Medical oncologist
	“I suppose it would be the training that I had from my mentors when I was a fellow. I always admired the way that they were able to be honest and up-front with patients, but not to scare them, or give them more anxiety than they already could have. That’s in terms of discussing risk, and then, in terms of a plan moving forward, I always like to have those going hand-in-hand, I think talking with our AYA group here, and getting a better understanding of what resources there are available for patients has been very informative.”	Medical oncologist

All oncologists who reported awareness of oncofertility guidelines reported that they informed their fertility discussions. Their use ranged from referring to guidelines “occasionally, but not often [referencing guidelines in discussions]” (Medical oncologist) to “we routinely use those [guidelines] to guide us” (Medical oncologist).

Scientific literature was reported by several oncologists as informing their fertility discussions with patients. One stated,

“If I’m planning on having the discussion with each patient and then somebody demonstrates interest, I go back and again do a bit of a literature review. From a fertility standpoint for chemotherapy, you usually have to do an updated literature review.” (Medical oncologist)

Only two oncologists stated that their experience and/or training informs their fertility discussions, highlighting the limited formal training surrounding impacts to fertility after cancer treatment received by providers. One added,

“I suppose it would be the training that I had from my mentors when I was a fellow. I always admired the way that they were able to be honest and up-front with patients, but not to scare them, or give them more anxiety than they already could have. That’s in terms of discussing risk, and then, in terms of a plan moving forward, I always like to have those going hand-in-hand, I think talking with our AYA group here, and getting a better understanding of what resources there are available for patients has been very informative.” (Medical oncologist)

### Quantitative findings

AYAs (*N* = 58) were, on average, 32.0 years at diagnosis (SD = 5.3; range 19–39), female (53.5%), Hispanic (35.1%), White (58.9%), college graduates (34.5%), with an annual household income of $80,000+ (57.2%; Table 4). All were insured, either privately (86.2%) or publicly (13.8%). AYAs were diagnosed with stages I (22.4%) and II (25.9%) diseases; 31.0% reported not knowing or being unsure of their disease stage (this may be because staging was collected at time of recruitment and patients could still be in the early phase of receiving diagnostic information). AYAs were treated with surgery (53.5%), chemotherapy (41.4%), and radiation (25.9). On average, AYAs received 1.6 (SD=1.2) treatment modalities. Over half (58.1%) reported normal menstruation and one quarter (25.9%) reported a live birth prior to their cancer diagnosis. Premature menopause and surgery to remove one or both ovaries were reported by 22.6% and 16.1% of females, respectively. Low sperm count and surgery to remove one or both testicles were reported by 3.7% and 44.4% of males, respectively.

**Table 4:** Quantitative sample characteristics among AYAs (*N* = 58)

	***N* (%) or *M* (SD)**
**Sociodemographic factors**	
**Age at diagnosis** (range: 19–39)	32.0 (5.3)
**Sex**	
Female	31 (53.5)
Male	27 (46.5)
**Ethnicity**	
Hispanic	20 (35.1)
**Race**	
White	33 (58.9)
Asian American	10 (17.9)
Black/African American	2 (3.6)
American Indian/Alaska Native	2 (3.6)
Mixed race	6 (10.7)
Other	3 (5.4)
**Highest level of education**	
Some high school or less	2 (3.5)
Highschool graduate	10 (17.2)
Community college/trade school	6 (10.3)
Current college student	6 (10.3)
College graduate	20 (34.5)
Graduate school	14 (24.1)
**Annual household income**	
Less than $39,000	15 (26.8)
$40,000 to $79,000	9 (16.1)
$80,000 to $119,000	16 (28.6)
$120,000 or more	16 (28.6)
**Health insurance**	
Privately insured	50 (86.2)
Publicly insured	8 (13.8)
**Clinical factors**	
**Cancer type**	
Reproductive	17 (29.3)
Thyroid	11 (19.0)
Brain/spinal cord	9 (15.5)
Colorectal	7 (12.1)
Breast	6 (10.3)
Lymphoma	4 (6.9)
Other	4 (6.9)
**Stage**	
I	13 (22.4)
II	15 (25.9)
III	8 (13.8)
IV	4 (6.9)
Do not know/not sure	18 (31.0)
Treatment*	
Chemotherapy	24 (41.4)
Surgery	31 (53.5)
Radiation	15 (25.9)
Steroids	9 (15.5)
Immunotherapy	8 (13.8)
Hormonal therapy	5 (8.6)
CAR-T therapy	0
Targeted cell therapy	0
Stem cell transplant	0
**Treatment intensity**	1.6 (1.2)
**Fertility factors**	
Reproductive history prior to cancer diagnosis*	
Previous live birth	15 (25.9)
Previous miscarriage	8 (13.8)
Normal menstruation^a^	18 (58.1)
History of infertility	3 (5.2)
Adoption	0
**Have children**	18 (31.6)
**Fertility-related late effects**	
Experienced infertility as a result of cancer treatment	2 (3.9)
Surgery to remove one or both testicles^b^	12 (44.4)
Low sperm count^b^	1 (3.7)
Premature menopause^a^	7 (22.6)
Surgery to remove one or both ovaries^a^	5 (16.1)
Monthly menstrual cycle (period)^a^	
Stopped after cancer treatment	7 (23.3)
Did not stop after cancer treatment	15 (50.0)
Not sure if it stopped	5 (16.7)
Not applicable (I did not have a menstrual cycle before cancer)	3 (10.0)
Menstrual cycle did not return after cancer treatment^a,c^	6 (100.0)

^*^Participants could select more than one response

^a^Among females only (*N* = 31)

^b^Among males only (*N* = 27)

^c^Among those who reported that their monthly menstrual cycle stopped after cancer treatment

Overall, 82.6% (*N* = 33) of AYAs reported being informed of the impact of cancer treatment on their fertility, with 66.7% reporting that these discussions occurred at “some visits” (Table 5). AYAs who discussed fertility reported being very (39.4%) or moderately (27.3%) satisfied with their discussion. Most (63.6%) AYAs discussed fertility with their oncologist, and few discussed it with a physician assistant or nurse practitioner (12.1%), gynecologist (6.1%), or social worker (3.0%).

**Table 5:** Fertility discussion

	***N* (%)**
Informed of impact of cancer treatment on fertility prior to beginning treatment*	
Yes	33 (84.6)
No/not sure	6 (15.4)
How often were you informed of the impact of cancer treatment on fertility**	
At every visit	3 (10.0)
At a majority of visits	7 (23.3)
At some visits	20 (66.7)
Level of satisfaction with the fertility discussion received**	
Very satisfied	13 (39.4)
Moderately satisfied	9 (27.3)
Neither satisfied nor dissatisfied	5 (15.2)
Moderately dissatisfied	3 (9.1)
Very dissatisfied	0
Not applicable (I did not need fertility preservation counseling)	3 (9.1)
Who informed you of the impact of cancer treatment on your fertility**^a^	
Medical oncologist	21 (63.6)
Physician assistant or nurse practitioner	4 (12.1)
Primary care doctor	2 (6.1)
Gynecologist	1 (3.0)
Social worker	0
Other	5 (15.2)

^a^Participants could select more than one response

^*^N = 16 reported not applicable and were excluded from analyses

^**^Among patients who reported a fertility discussion

AYAs who reported a fertility discussion reported “rarely” or “never” discussing the following components with their provider: (1) a history of cancer, cancer treatment, or fertility treatment does not increase the risk of cancer or birth defects in your child, except in special circumstances (69.7%); (2) provided with information to patient advocacy resources (for example, FertileHope, Livestrong, the Susan G. Komen® Breast Cancer Foundation, and the Colon Club; 66.7%); (3) offered a referral to supportive counseling prior to treatment with adequate time to use these resources (63.6%); (4) there is no increased risk of cancer returning because of preserving fertility and pregnancy (60.6%); (5) if you are female, timing of fertility treatments based on your menstrual cycle (period; 40.0%); (6) individual factors such as disease, age, treatment type and dosages, and pre-treatment fertility (33.3%); and (7) considering your options as soon as possible to increase the likelihood of success (33.3%, Table 6).

**Table 6:** Components of fertility counseling among AYAs who reported a discussion (*N* = 33)

Components of ASCO guidelines	Overall frequency with which components of ASCO guidelines were included in discussions						Aggregated total	
	**Always**	**Usually**	**Sometimes**	**Rarely**	**Never**	**I did not/do not have a menstrual cycle**	**Always/usually/sometimes**	**Rarely/never**
	*N* (%)	N (%)	*N* (%)	*N* (%)	*N* (%)	*N* (%)	*N* (%)	*N* (%)
Individual factors such as disease, age, treatment type and dosages, and pre-treatment fertility	6 (18.2)	7 (21.2)	9 (27.3)	5 (15.2)	6 (18.2)		22 (66.7)	11 (33.3)
Considering your options as soon as possible to increase the likelihood of success	8 (24.2)	4 (12.1)	10 (30.3)	2 (6.1)	9 (27.3)		22 (66.7)	11 (33.3)
There is no increased risk of cancer returning because of preserving fertility and pregnancy	4 (12.1)	4 (12.1)	5 (15.2)	1 (3.0)	19 (57.6)		13 (39.4)	20 (60.6)
A history of cancer, cancer treatment, or fertility treatment does not increase the risk of cancer or birth defects in your child, except in special circumstances	2 (6.1)	3 (9.1)	5 (15.2)	4 (12.1)	19 (57.6)		10 (30.3)	23 (69.7)
Offered a referral to supportive counseling prior to treatment with adequate time to use these resources	2 (6.1)	4 (12.1)	6 (18.2)	3 (9.1)	19 (54.6)		12 (36.4)	21 (63.6)
Provided with information to patient advocacy resources	4 (12.1)	3 (9.1)	4 (12.1)	1 (3.0)	21 (63.6)		11 (33.3)	22 (66.7)
Timing of fertility treatment based on your menstrual cycle (female only; *N* = 15)	5 (33.3)	1 (6.7)	0	0	6 (40.0)	3 (20.0)		

## Discussion

In this mixed-methods study at an NCI-designated Comprehensive Cancer Center, most oncologists shared that they did not have formal oncofertility care delivery training, which was not offset by other team members. Relatedly, oncologists’ awareness of guidelines for fertility discussion was low, and components of ASCO guidelines were infrequently included in fertility discussions. Despite this, AYAs reported moderate to high satisfaction with received counseling. While oncologists reported largely positive perceptions of guidelines for fertility discussion, several shared that guidelines themselves may hinder integration in clinical practice if they do not capture diverse patient scenarios or scientific advancements and/or are outdated. Ultimately, refining guidelines to optimize counseling recommendations and subsequently improving their integration in clinical practice may ensure AYAs are provided with desired and actionable information to make goal-concordant reproductive decisions.

Most oncologists reported that they did not have training surrounding oncofertility care delivery, and this lack of training was not offset by other team members, suggesting a systemic issue and highlighting a gap in medical education. Notably, at the time of data collection in 2023, participating oncologists were on average in practice for over 14 years. Thus, it is possible that oncofertility was not included in their medical curriculum as the first iteration of ASCO clinical practice guidelines was published in 2006 and may not yet have been integrated into training [6]. In the present study, all oncologists who were aware of guidelines reported that they informed their fertility discussions. However, this is in contrast to prior research in which 44% of pediatric oncology care team members were familiar with ASCO oncofertility guidelines, with only 39% reporting using these guidelines in practice [43]_._ As this study followed the initial release of guidelines in 2006 and ASCO has since published three updated iterations of oncofertility counseling guidelines (in 2013, 2018, and 2025), it is possible that use may be increasing among oncologists who report awareness [6–9]. Ultimately, given the widespread use of gonadotoxic therapies for cancer patients and the importance of reproductive health to AYAs [14, 44], if not already included, training on oncofertility warrants greater integration into the medical curriculum and postgraduate training. In an international study defining key components of oncofertility care, 9 in 10 health care professionals (including oncologists, nurses, endocrinologists, oncofertility staff, among others) and patient and family advocates endorsed that health care professionals should have knowledge of oncofertility guidelines, and most agreed or strongly agreed that staff familiarity with national and/or international oncofertility guidelines is an essential component of oncofertility services [45]. Relatedly, staffing a designated patient navigator with training in oncofertility has been found to increase the frequency of counseling, improve patient satisfaction, and may support patients and clinicians alike by offsetting limited knowledge among oncologists [46–48]. In addition, continuous institutional investment in physician education is needed through accessible avenues such as continuing medical education (e.g., requiring oncofertility training modules for oncologists treating AYAs) and tumor boards to ensure that oncologists maintain an adequate and current threshold of competency to properly inform patients of possible fertility-related implications of cancer and its treatment. Overall, efforts to increase provider and thereafter patient knowledge surrounding oncofertility are needed to ensure AYAs are provided with comprehensive counseling.

Among AYAs who received fertility counseling, most reported discussions with their oncologist, and the majority was moderately or very satisfied with the information received. However, oncologists’ awareness of fertility discussion guidelines was low, and AYAs reported only two components of ASCO guidelines that were included in over half of discussions (i.e., discussing individual factors and the timely consideration of fertility preservation). Despite this, overall satisfaction with discussions among AYAs was moderate to high, suggesting adherence to all components of guidelines may not be required for patient satisfaction. Prior research among patient desires for oncofertility counseling revealed that information about fertility status, contraception, safety of pregnancy, alternatives to biological parenthood; help managing pressure, cost, uncertainty, and navigating the healthcare system; and detailed, accurate, and understandable information are important to patients [29, 49–51]. However, research on the content of fertility discussions is infrequent, and we currently have a limited understanding of what discussion components are seen as actionable by patients. Therefore, future research aimed at better understanding counseling, its concordance with patient information-related preferences, and how these preferences align with guidelines may inform the refinement and prioritization of guidelines to optimize oncofertility counseling recommendations.

Some oncologists reported that guidelines may not encompass diverse patient scenarios, limiting alignment with the more personalized focus of contemporary cancer care delivery, and others perceived guidelines to be outdated or not reflective of ongoing scientific advancements, hindering their applicability to clinical practice. These perceptions of guidelines may account for the limited guideline-concordance reported by AYAs. In a prior study, clinicians reported limited agreement with ASCO guidelines as infertility was perceived as secondary to cancer treatment, an interference with treatment, and not a life-threatening concern [52]. Similar to the perspectives of oncologists in the present study who shared concerns regarding the applicability of these guidelines in practice, the 2013 iteration of the ASCO guidelines (second version, most recent at time of the Font-Gonzalez et al., 2016 study) was determined to be high-quality in accordance with the Appraisal of Guidelines for Research and Evaluation II instrument (AGREE II) [53]; however, the lowest domain was applicability, and the highest was clarity of presentation [54]. Though limited research surrounding the monitoring of guidelines exists, some suggest that clinical practice guidelines should be reassessed every three years [55]. Nevertheless, dedicated efforts by professional organizations are needed to commit to the regular and consistent update of guidelines to ensure ongoing advancements in care are incorporated. To remedy challenges with guideline integration at the organization level, institutions may consider developing a decision-tree framework unique to their care setting to simplify oncofertility care delivery into applicable and manageable step-by-step parts with various pathways to account for varying patient care needs [56]. In aggregate, findings suggest that challenges implementing oncofertility guidelines may not be limited to provider-specific barriers but may also reflect barriers native to guidelines themselves that require modification for increased adherence.

This study has several strengths and some limitations. To our knowledge, this is the first mixed-methods study to examine the uptake of ASCO oncofertility guidelines in practice from the perspectives of AYA patients and oncologists, to delineate patterns from the perspectives of oncologists regarding challenges they may face implementing these guidelines, and to map received counseling onto ASCO guidelines in a detailed manner. Study findings also provide novel insights regarding how oncofertility guidelines are or are not used by oncologists in a “best case scenario” setting such as at an NCI-designated Comprehensive Cancer Center (academic medical center). These findings are strengthened through the combination of research methods that provide a comprehensive overview of perspectives from both patients and oncologists. However, as oncologists are the treating physicians, are widely believed to initiate fertility discussions, and as AYAs rank oncologists second when asked their preferred source of information for learning about oncofertility (61% of AYAs endorsed fertility specialists, 55% oncologists, 33% nurses) [20, 21, 30]_,_ we did not interview different members of the cancer care team that may be involved in fertility counseling. Further, we were unable to recruit oncologists of all specialties that frequently treat AYAs, such as hematologist-oncologists. In addition, as limited research involving the use of guidelines in practice exists, we sought to detail this experience in a single care setting, though this limits generalizability to other settings. Lastly, though responding AYAs were within 6 months of diagnosis at the time of the survey, there is a risk of recall bias as data were collected via self-report survey and fertility counseling sessions were not directly observed, yielding a potential method for future data collection.

Provision of counseling by both fertility specialists and oncologists may result in lower decisional regret than provision of counseling from oncologists alone, and we note this as an important integration into care delivery pathways where resources permit [20]. Future research may build on these findings by recruiting from the multidisciplinary cancer care team, understanding what components of guidelines are seen as actionable by AYAs, and ultimately developing interventions to increase oncologists’ knowledge of guidelines. In parallel, clinical practice organizations should continue regular revision and update of oncofertility guidelines using community-engaged and participatory methods to develop patient-centered guidelines, increase applicability of guidelines to AYAs’ preferences, and ensure that oncologists are provided up-to-date guidance on optimal counseling.

## Conclusion

As recently as March 2025, a new iteration of ASCO guidelines was released with revised counseling recommendations, risks of infertility, fertility preservation protocols, and the role of clinicians in care delivery [9]. Such oncofertility guidelines are intended to improve patient care, but barriers to their integration in clinical practice may compromise their effectiveness. Understanding factors that impede guideline uptake can inform future interventions to ensure AYA cancer patients receive recommended supportive care. Though limited knowledge and training surrounding oncofertility guidelines among oncologists and the stringent or outdated nature of guidelines may compromise the delivery of comprehensive reproductive health care, both are amenable to targeted interventions to facilitate informed decision making among AYAs. As all components of guidelines may not be necessary for patient satisfaction with discussions, understanding patient information-related preferences may inform the refinement of guidelines to optimize oncofertility counseling recommendations. Subsequently, improving their integration in clinical practice may ensure AYAs are provided with desired and actionable information to make goal-concordant and well-informed decisions for their reproductive futures.

## Supplementary Information

Below is the link to the electronic supplementary material.

Supplementary file1 (DOCX 23 KB)

## Data Availability

The data that supports the qualitative findings of this study are available in text or supplemental material of this article. Quantitative data are available upon request.
