# Supplementary material for: Are guidelines guiding? A mixed methods study examining the integration of ASCO fertility discussion guidelines in practice among oncologists and adolescents and young adults at an NCI-designated Comprehensive Cancer Center
Source: J Cancer Surviv. 2025 Jun 19;20(Suppl 1):663–88. doi: 10.1007/s11764-025-01850-0 (PMC13008573; doi:10.1007/s11764-025-01850-0)
Supplement: Supplementary file 1 [file 11764_2025_1850_MOESM1_ESM.docx]

| **Supplemental Table 1:** Oncologists training surrounding oncofertility | | |
| --- | --- | --- |
|  | **Quote** | **Oncologist** |
| **No formal training** | "That’s a fair question. I can’t say I am. I’m sure there’s a lot I don’t know, or we don’t get routine training or updates or anything like that officially." | Medical oncologist |
|  | "Training? Gosh. I don't think anybody's had any training." | Medical oncologist |
|  | “I don’t think any of us have formal training. I guess in my fellowship training we certainly had lectures and such, but I don’t think…. Do you mean our nurses and our PAs? I don’t think they get any formal training. Though, of course, the social workers do and we used to work closely with our AYA social worker who left [institution] a few months ago. I think they just hired a replacement, so I’m sure we’ll be working closely with [them]. I’m sure they have formal training there.” | Medical oncologist |
|  | "None within my team specifically. I’m a testis cancer expert, so that’s part of it but not specific expertise in infertility. Let’s say we get the semen analysis back and it’s very low sperm count, then I’ll refer to the specific person within my group, [provider], who is a fertility specialist." | Medical oncologist |
|  | “[LAUGHS] That’s a good question…I certainly don’t have any certifications or licenses specific to it. I do have training regarding side effects and potential side effects related to treatments. So, in that regard, I would say that I have some, but I think if it’s anything more specialized than that, no we don’t have anybody, not directly on my team at least.” | Medical oncologist |
|  | "Now, in my practice, because I also use some newer, more specialized treatments, I think the onus is on me to be well-informed about what are the risks with each of those drugs. That’s not something that I really have a lot of training on, but it is something which I do, in the same way that I would with any new drug and any potential side effect." |  |
|  | "That’s a good question. I don’t know if any of us have actual formal training in fertility or reproductive health." | Medical oncologist |
|  | “I don’t know that anybody truly has training. It’s just me and her [nurse practitioner]. I don’t know that she’s done any extra training for it in her studies. As far as my studies go, there’s not something that’s taught to us in med school as far as chemotherapy infertility other than to say that it causes infertility. So, I don’t know there’s any special courses that at least I haven’t gone through.” | Medical oncologist |
|  | "I think nobody. Nobody has formal training." | Medical oncologist |
| **Experience** | "Just by experience…" | Medical oncologist |
|  | "I think we just learn as we do it." | Gynecologic oncologist |
|  | “Specific training for reproductive health or fertility, we just learn as we go.” | Medical oncologist |
| **Lectures during training** | "When I’m referring to lectures, we had awkward fertility lectures in fellowship and we have them currently on our fellowship syllabus, where one of the gynecologists from the [institution's] fertility clinic comes and lectures our fellows and talks to them about that. I don’t recall the specifics." | Medical oncologist |
|  | "When we were doing rotations in training. So, I don’t think we have any specialized education, but that would probably be very informative and helpful, especially for the follow-up questions that our patients sometimes ask." | Medical oncologist |
|  | "Beyond what we learn in medical school or what we look into on our own literature review, and I don’t know about the AYA team’s formal training. I can’t speak to that." | Medical oncologist |
| **Colleagues or mentors** | "Having conversations with other practitioners. I always get tips, or someone says ‘Oh, I found this great resource.’ So just discussions with other people, collaborations with other specialties.” | Medical oncologist |
|  | "I lean on my mentors and colleagues. My local resources, and then also my national and international resources. So, mentors and colleagues that I work with and whom I’ve trained with, they’ve kind of set the foundation for how I approach the discussion." | Medical oncologist |
| **Gynecologic oncologists have training** | "It's something that we just do all the time and that I've been doing since residency. So, I teach discussing it more than ongoing learning about it." | Gynecologic oncologist |
|  | "Just the physicians [have training], like me and my partners if they were covering for me, but not any of the other nurses or mid-level providers." | Gynecologic oncologist |

| **Supplemental Table 2:** Oncologists' awareness of fertility discussion guidelines | | |
| --- | --- | --- |
|  | **Quote** | **Oncologist** |
| **Reported guidelines** | “Not anything above what I've mentioned. Things that are major announcements at meetings, or articles that come out that get a lot of press. NCCN guidelines are always useful.” | Medical oncologist |
|  | “I'm sure there is stuff that ASCO has. I'm sure there are things that NCCN has." | Gynecologic oncologist |
|  | "The NCCN has some guidelines out there and ASCO has some guidelines on fertility discussions as well…So for major organizations, they have guidelines for that." | Medical oncologist |
|  | "I think the different associations will have guidelines, whether it’s ASCO or NCCN or AYA resources and then every cancer center will have their own protocol as well." | Medical oncologist |
|  | "That I am aware of, I don’t know, other than the AUA testis cancer guidelines, which mentions fertility. I don’t think the prostate cancer guidelines do and certainly not the bladder cancer guidelines because of the age range of patients it's a minor, minor, very, very rare situation." | Medical oncologist |
|  | "The ASCO guidelines and then other major clinical bodies, American Society of Hematology, the European Society of Medical Oncology, and the Society for Immunotherapy and Cancer. Those are probably the main ones. Oh, I’m sorry, the NCCN as well." | Medical oncologist |
|  | "NCCN definitely has guidelines for fertility-sparing options for each of the GYN cancers. SGO definitely has, and ACOG, definitely have materials about it. I don’t think they have separate specific guidelines per se and I know ASCO has guidelines, but in the GYN cancers, I tend to use NCCN more." | Gynecologic oncologist |
|  | "I think that there are some NCCN guidelines related to this, but I’m not 100% sure." | Medical oncologist |
| **Unaware of guidelines** | “I'm not sure of the guidelines that are specifically in place, other than that it's a good thing to mention and to offer counseling on it. I'm sure there are guidelines, but I'm not familiar with them." | Medical oncologist |
|  | "I guess I would have to review them first because I don’t know." | Medical oncologist |
|  | "I know as fellows, we really fixate on the young testicular. So, maybe that’s why I lean towards that and obviously any treatment that has adverse events with fertility, but otherwise, I would say not that I’m aware of, but I’m sure there are." |  |
|  | "Honestly, I have never read them. So, I am going to be totally honest. I assume they exist, but I have never seen them.” | Gynecologic oncologist |
|  | "I don’t know. I haven’t really used external ones." | Medical oncologist |
|  | "My short answer is no, only because I’m not really aware of any guidelines specifically geared toward brain tumors, or fertility and brain tumors in AYA, but I would be very open, I think that it just means that’s a great place for further investigation for our needs and how we can meet them for our patients." | Medical oncologist |
|  | "Again, because the general guidelines, or general oncology is so different from neurooncology, there are none that I’m aware of, in terms of general non-brain-tumor specific, but I’m sure that there are some that are out there, again, because it’s such an important topic." |  |
|  | "I’m not aware of any guidelines or recommendations on who should be addressed.…So there’s no true guideline that I follow. If they’re young and they have infertility [as a] possibility, then I would talk it over with them." | Medical oncologist |
|  | “I don’t know. [LAUGHS] Maybe you could tell me. I’m not aware of any guidelines on fertility discussions. Maybe it’s more prevalent in the GYN oncology world but for medical oncology I’m not aware of guidelines for fertility discussion.” |  |
|  | "I think there are some guidelines. I don’t know them off the top of my head, but I have seen some literature [INDISTINCT] infertility associated with certain chemotherapy agents that I’ve looked through in the past." | Medical oncologist |
|  | "I think admittedly I’m not as familiar with the algorithms or the guidelines as I should be." |  |
|  | I don’t know if there are clear national cancer guidelines, hopefully, now I’m gonna go and look into it. [LAUGHS] |  |
| **Lack of knowledge hinders discussions** | "I don’t know specifics in terms of cost, so I can’t be the one to do it." | Medical oncologist |
|  | “I don’t know the data all that well either, so I give them a rough estimate, but I think that when I’ve searched in the past, the studies that I’ve seen aren’t really specific to any chemotherapy drugs, so sometimes patients will say, ‘What percentage do I have of getting pregnant after if I do chemotherapy?’ And I haven’t really seen excellent, clear, randomized trials obviously, or really great retrospective studies. So, I give them my estimate [of infertility risk] with the limitation I think there’s not great data to support it, but to understand with the assumption that it’s less than before.” | Medical oncologist |
